# The relationship between subconstructs of empathy and general cognitive ability in the context of policing

**DOI:** 10.3389/fpsyg.2022.907610

**Published:** 2022-11-23

**Authors:** Miguel Inzunza, Gavin T. L. Brown, Tova Stenlund, Christina Wikström

**Affiliations:** ^1^Police Education Unit, Faculty of Social Sciences, Umeå University, Umeå, Sweden; ^2^Faculty of Education and Social Work, The University of Auckland, Auckland, New Zealand; ^3^Department of Applied Educational Science, Faculty of Social Sciences, Umeå University, Umea, Sweden; ^4^Department of Psychology, Faculty of Social Sciences, Umeå University, Umeå, Sweden

**Keywords:** cognitive empathy, policing, general cognitive ability, latent variable model, subconstructs

## Abstract

**Purpose:**

Empathy has been widely theorized as an important ability in professions such as policing, in which to perform well individuals require multiple and interacting abilities, not least when resolving conflict situations. Even so, there are few studies investigating how subconstructs of empathy relate to other constructs such as general cognitive ability. The purpose of this paper is to establish, after evaluating psychometric properties, relationships among measures of empathy and cognitive ability in a sample of Swedish police students (*n* = 157).

**Design/methodology/approach:**

Multiple latent variable models of how the different measures work to predict tasks that can be seen as proxies for the ability to understand another person’s situation and intentions are evaluated to determine the most robust relationship(s) within the data.

**Findings:**

We find support for the psychometric properties reported in previous studies with the used instruments. We also find support for perspective-taking, a cognitive empathy subconstruct predicting the ability to recognize emotions, and also the affective part of empathy, predicting general cognitive ability. These findings are discussed at length in the paper.

**Originality/value:**

This research adds more knowledge to the issue of how general cognitive ability relates to cognitive empathy and other subconstructs of empathy or Theory of Mind.

## Introduction

The ability to understand others’ situations and intentions (i.e., empathy or Theory of Mind (ToM)) involves being able to perceive events or phenomena from the point of view of another person (Baron-Cohen et al., 2015). Possessing this capacity is important when dealing with difficult situations, such as when dealing with a victim or a suspected perpetrator at a crime scene (Beauregard et al., 2007; Maddox et al., 2011) or when trying to de-escalate a conflict (Abanonu, 2018).

Understandably, empathy and/or ToM is associated with general cognitive abilities (Gore et al., 2010; Rakoczy et al., 2012). However, there is little evidence of how empathy relates to higher-order constructs, such as intelligence, because it may have more to do with memory, that is if the observer has previous experiences of what a person might be experiencing in specific situations (Baron-Cohen et al., 2001). Hence, this paper examines the relationship of empathy to a number of related mental constructs (i.e., fluid intelligence, emotion recognition, intention detection, and working memory) in the context of police education, which emphasizes the importance of empathy. The study contributes to the field by showing that affective empathy has more to do with fluid intelligence, while cognitive perspective-taking related to emotion recognition.

### Understanding empathy

Research into the process of understanding another person’s intentions or experiences as a facilitator of daily encounters can refer to related processes (Baron-Cohen et al., 2015; Launay et al., 2015). Cognitive empathy, perspective-taking, or ToM are terms used to describe these processes (Baron-Cohen et al., 2015; Launay et al., 2015). However, empathy has two dimensions, one cognitive and one affective (Decety and Jackson, 2006; Jolliffe and Farrington, 2006). The cognitive dimension involves understanding another person’s intentions or experiences and beliefs of others (Singer, 2006). The affective dimension includes the bodily sensation such as being able to mimic the expression of others’ emotional states (Decety and Jackson, 2006). While rapport has been suggested as an essential component of interpersonal relations (Jakobsen, 2021), it is not clear whether this involves cognitive or affective components.

This means that cognitive intellectual abilities involved in empathy may not be sufficient to explain why or how empathy operates. The number of studies focusing on the relation between empathy and cognitive ability is limited. An early study used the revised version of the *Reading the Mind in the Eyes test* (RMET), which measures how a test-taker can understand the mental state of another person (Baron-Cohen et al., 2001). The study reported no statistically significant correlation between the RMET and IQ (*r* = 0.09, *p* = 0.60). However, a later meta-analysis investigating how the RMET correlated to intelligence found a positive significant correlation (*r* = 0.24) with a robust effect size (Baker et al., 2014).

Launay et al. (2015) found that there was a positive association between higher forms of mentalizing and emotion recognition in the RMET (*r* = 0.21, *p* < 0.001, *n* = 279), but they did not find any relation between mentalizing scores and self-reported empathy, measured with the *Empathy quotient* (EQ) instrument (*r* = 0.022, *p* = 0.33, *n* = 282). Guo et al.’s (2019) study with undergraduate students (*n* = 518) reported a significant correlation between perspective-taking in the *Interpersonal Reactivity Index* (IRI) and fluid intelligence measured with *Raven’s Standard Progressive Matrices*, but the correlation was stronger between affective empathic concern and fluid intelligence (*r* = 0.17 vs. *r* = 0.27). In a multiple mediation analysis, they reported cognitive ability had a positive effect on perspective-taking (*β* = 0.22, SE = 0.03, *p* < 0.001) and also on empathic concern (*β* = 0.26, SE = 0.03, *p* < 0.001).

From this research, it would appear that the affective aspect of empathy is dependent on overall intelligence, but self-reported cognitive perspective-taking may be quite independent. However, further research with normal populations is needed to establish the nature of empathy outside clinical groups (Richell et al., 2003).

A related capacity to intelligence that may be related to empathy is executive functioning (EF). Yan et al. (2020) found a significant correlation between cognitive empathy and subcomponents of EF such as inhibitory control (*r* = 0.23, *p* < 0.001) and working memory (*r* = 0.20, *p* < 0.001) was found, while the affective dimension of empathy had a weaker relation. Another recent meta-analysis (Martingano and Konrath, 2022) found a positive association between the cognitive part of empathy and rational thinking as expected with both empirical data and meta-analysis.

Another issue related to the measurement of empathy is the difficulty individuals have in providing valid information on their empathic ability (Murphy and Lilienfeld, 2019). Murphy and Lilienfeld (2019) suggest that the weak relations between empathy-related self-reports and task performance might be a consequence of measures that do not have acceptable measurement quality. In line with these reflections Baker et al. (2014) argue that the correlation would probably be even stronger if tests measuring these types of constructs had levels of reliability that were as high as intelligence tests.

### Empathic policing

Empathy has been identified as an important ability when working as a police officer (Perez, 2010; Inzunza, 2015a). It is particularly relevant for professions where interaction with the public often involves difficult circumstances (Skogan, 2006; Rahr and Rice, 2015; Inzunza, 2015a). In the field, a police officer needs to understand how others perceive a complex critical situation, and at the same time have the capacity to regulate their own perceptions in order to act professionally. When developing models that include these types of processes they are often multi-dimensional models that include both a cognitive and an affective dimension (Davis, 1983; Inzunza, 2015b). The importance, and indeed the necessity, of including other elements such as regulating processes has also been discussed and incorporated in some of these models. This is especially relevant when applied to professions such as policing, where the complexity of some daily encounters may require robust self-regulatory processes (Inzunza, 2015a). In this context, the major goal is to end encounters without harm for any party. This requires the ability to predict how a situation may develop based on the various actors’ intentions in a social situation (Abanonu, 2018).

Policing, therefore, requires an adequate level of intelligence and resilience (Romosiou et al., 2019), as well as both cognitive and affective empathy. This perspective-taking and empathy capability is important for police, especially when conducting interrogations. There are several ways offenders act to make a story sound truthful (i.e., offender lie-telling strategies; Strömwall and Willén, 2011). Such strategies can involve impression management strategies or information management strategies, both of which require close attention from the police interrogator. Interrogators need to pay attention to minute details and use their verbal communication skills to retrieve information so that they can understand the intentions of the offender. Here the primary goal is to increase the probability of solving complex crime events. All parts are necessary when approaching a crime within the framework of the choices and decisions made by alleged offenders before, during, and after committing a specific type of crime (Leclerc, 2017).

Police need to demonstrate empathy also when working with crime victims (e.g., Maddox et al., 2011; Inzunza, 2022). This seems to be a somewhat challenging ability to learn for police students because the identity formation process that takes place within the profession struggles to treat vulnerable citizens appropriately (Charman, 2019).

A small-scale (*n* = 60) study with police students found no statistically significant relations when studying the total scores between a subjective self-report measure of empathy (i.e., an adapted version of the Empathy Assessment Index; EAI) and objective tasks aimed at measuring similar constructs (Inzunza et al., 2019). However, those self-reporting highly on perspective-taking also performed better on the objective tasks. Other studies have noted the importance of understanding the relation of subconstructs of empathy to other constructs.

The practical value of researching this area can be seen both in the context of selection and the education and training of future police officers. The construct of empathy has been identified as crucial for general interactions in terms of social skills but also when conducting training in specific units within police organizations such as de-escalating conflict situations (Abanonu, 2018). In a recent Danish study of how official political goals have been translated to measurable criteria, the construct of empathy played a crucial role (Bloksgaard and Prieur, 2021). The study concluded that, within Danish police, police officers need to express emotions in a controlled way so that these communicative skills counterbalance their authority and ability to act; indicating the importance of empathy in modern policing.

Training of police is an important tool to their development of de-escalating strategies. Performance associated with empathy (e.g., understanding the situation of the other) is at focus when implementing specific programs aimed at improving police officers’ interviews with victims (Darwinkel et al., 2013).

A more detailed understanding of how aspects of empathy relate to other cognitive abilities is important to the design of police education programs. Hence, this study focuses on empathy and its subconstructs, how they can be measured and how they relate to other selected measures within police education populations. Two hypotheses were investigated:

*H1*: There is a relation between subconstructs of empathy and cognitive ability.

*H2*: Subconstructs of empathy have a positive relation to tasks measuring the corresponding abilities.

## Materials and methods

Thus, the goal of this paper is to establish if there are relationships between measures of empathy and cognitive ability in a sample of Swedish police students. A prioritization was to collect information from the population of interest and support the validity argument of presenting a valid interpretation of the results from this specific group of test-takers (Kane, 2010). A key feature of this study is that it integrates self-reports and objective tasks. Multiple models of how the different measures work to predict tasks that can be seen as proxies for the ability to understand another person’s situations and intentions are evaluated to determine the most robust relationship(s) within the data.

### Participants

The participants in this study were police students enrolled in three of the Swedish national police programs during fall of 2017 (i.e., Umeå, *n* = 54; Södertörn, *n* = 48; and Linnaeus, *n* = 55). The approximate percentage of males was 72–73%, and their mean age in years was 25.7 (Umeå), 26.2 (Södertörn), and 25.4 (Linnaeus). Compared to the total group of police students in Sweden, this group of 157 students is relatively representative in terms of age and gender distribution (polistidningen.se). Through voluntary participation requirements, the obtained response rate varied by location (Umeå, 87%; Södertörn, 22%; and Linnaeus, 22%). The amount of police students differs between the three locations where Södertörn and Linnaeus have more students enrolled in their programs and it is more difficult to reach all students. Since the allocation of Swedish National Police students is centrally organized, the different police programs have a similar profile regarding individuals recruited, and they experience similar content throughout their training. It is noteworthy that Swedish police education is a two-year undergraduate university qualification plus 6 months on-the-job apprenticeship, rather than a brief sub-baccalaureate qualification as seen in some other jurisdictions. Of the 157 participants, complete data on all constructs ranged between 154 and 157, suggesting a very high rate of data recovery. Note that no pressure was put on the respondents to participate or to perform well on the different measures, meaning score means and relations might be different if the scores were used to select candidates for entry to the police.

### Instruments

A battery of instruments was deployed to measure subconstructs of empathy and cognitive ability. The three instruments used to measure empathy and ToM (i.e., Swe-EAI, Eyes test, and Swe-IMT) have all been used in the police context in previous studies (see Inzunza, 2015b; Inzunza et al., 2019). Intellectual ability was measured with the Raven APM Set II measures of fluid intelligence (also referred to as cognitive ability).

#### Empathy assessment index (Swe-EAI)

The Swedish version of the EAI (Swe-EAI) is a self-report instrument that examines test-taker perceptions of their own ability to empathize. The Swe-EAI differentiates four aspects of empathy (Inzunza, 2015b), being affective response (AR, with six items), perspective-taking (PT, with six items), self-other awareness (SOA, with four items), and emotion regulation (ER, with four items). Participants respond to the items by choosing between six alternatives on a scale from *never* to *always*. A previous study (Inzunza, 2015b) indicated that the four-factor model had acceptable fit and levels of reliability for the four dimensions. The reported estimate of internal consistency for the complete scale was *α* = 0.74, for the affective dimension with the AR factor *α* = 0.70, for the cognitive dimension including the factors PT, SOA, and ER *α* = 0.73 (Inzunza, 2015b). Since SOA and ER are part of the regulating system in the process of empathy (meaningful when all subscales are used to measure empathy, but not when making the distinction between affective and cognitive empathy) as proposed and discussed in a previous study by Inzunza (2015a), they were omitted from the inter-correlation and relationship analyses to the other measures.

#### Imposing memory task (Swe-IMT)

The Swedish version of the IMT (Swe-IMT) was initially developed to assess how respondents could deduce the intentions and actions of others by understanding their mental states—this is also referred to as *mentalizing* ability (Kinderman et al., 1998). This test consists of three short stories about social situations with several characters, in which the test-taker, after reading the stories during a short period of time, answers questions and statements in an answer booklet. The questions and statements, answered in a true/false format, measure either intention (ToM) or memory. The outcome of the test can be reported as a total score or as sub-scores for the intention items, the memory items, or the mean number of errors. In a previous study (Inzunza et al., 2019) with 78 Swedish national police students participating, the mean error for 30 intention items was 5.8 (*SD* = 2.5) and the mean error for the 36 memory items was 4.8 (*SD* = 2.5). The intention and memory scores had a statistically significant correlation to each other (Spearman’s *ρ* = 0.42, *p* < 0.01). To avoid fatigue, two stories rather than three were used, producing a test of 20 intention and 24 memory items.

#### Reading the mind in the eyes test (Eyes test)

The Eyes test requires the test-taker to infer the mental state of another person based solely on a picture of their eyes (Vellante et al., 2013). The underlying assumption of the test is that the mental state of a person can be determined by the appearance of their eyes. The test consists of 36 images of different persons expressing a mental state and a four-option multiple-choice questions for each image about the intention or emotion of the picture. Several previous studies with different populations have reported mean scores ranging from 26.2 (*SD* = 3.6) to 28.0 (*SD* = 3.5; Baron-Cohen et al., 2001; Söderstrand and Almkvist, 2012; Inzunza et al., 2019). Reliability in terms of alpha has not been frequently reported in the original version of the Reading the Mind in the Eyes Test; instead, there are examples of Guttman’ split-half with a value of 0.77 with the Eyes test or test–retest reliability by intraclass correlation coefficient at 0.833 (Vellante et al., 2013). Not reporting the alpha reliability has been the common procedure with tests designed to assess emotion recognition due to the complexity involved in those tasks when correlating different items (Fernández-Abascal et al., 2013). On the other hand, there are other benefits with this type of test. In contrast to self-reports, a test of this sort is insensitive to socially desirable responses (Van Prooijen and van Dijk, 2014). Another dimension is the distinction between reliability and homogeneity, where the latter is not helpful for validity when high (Cattell and Tsujioka, 1964). The main attention in previous studies has been given to investigating the validity of the test, i.e., to what degree it captures different mental states and expressions and measures the respondent’s abilities to distinguish between these.

#### Raven APM set II fluid intelligence ability (Ravens)

The Raven APM Set II measures the fluid intelligence ability to solve problems without reference to previous information or knowledge (Carpenter et al., 1990; Bors and Stokes, 1998). The items consist of matrices of eight related patterns presented across three rows, where the test-taker has to complete the matrix pattern by choosing the correct ninth option from a set of eight alternatives. The entire Set II consists of 36 problems which require approximately 40 min to complete. The reported mean score from a sample of 506 first-year university students was 22.17 (*SD* = 5.60), and reported internal consistency in terms of alpha was 0.84 (Bors and Stokes, 1998). In another study with university students, the reported mean score was 23.7 (*SD* = 5.7; Arthur Jr and Day, 1994). However, in this study, each participant answered just 18 items from either the odd or even numbered items to avoid fatigue. Given that items are of increasing difficulty this generates two parallel tests of fluid intelligence. This type of instrument was expected to be familiar to the participants in our research since intelligence tests are used in the selection procedure for applicants to the Swedish police program (Annell et al., 2014).

### Procedure

The police students that participated in this study were informed that their participation was voluntary in an oral introduction given at the police academies and also in a cover letter. The participants were assured that all data would be treated according to prevailing research practices regarding confidentiality and ethics. The same procedures were at work in each of the three universities where data were collected. The police students completed the battery of instruments as described earlier. Data were collected from groups at each academy, with the participants sitting at individual tables under invigilated examination conditions.

### Statistical analyses

Consistent with the two-step protocol (Anderson and Gerbing, 1988), measurement characteristics of each instrument were determined before inter-construct modeling took place. A combination of confirmatory factor analysis (CFA) and scale reliability estimation was used to establish the best-fitting set of items for each scale. Once scales were validated, inter-relationships among scales were examined using multiple structural equation models. Tested models included (a) simple inter-correlated scales, and (b) structural equation models (SEM). Analyses were conducted with latent variable software Mplus version 8 (Muthén and Muthén, 1998-2017). STATA version 16.1 (2020) was also used for calculating Raykov’s factor reliability coefficient.

*Swe-EAI*. Because the Swe-EAI has four latent factors, CFA was used to evaluate its structure.

*Swe-IMT, Eyes test, and Ravens*. These scales use classical test theory scoring protocols, summing all items scored as correct according to the scoring key and thereafter calculating reliability coefficients. The test and sub-test scores are evaluated for reliability with scale reliability estimation, which allows calculation of the standard error of measurement (i.e., SEm = SD*√(1–*α*)).

*Data preparation*. Because the sample size is small relative to the total number of items (155,118), SEM analysis used parceled scale scores for the Swe-IMT intention and memory, Eyes test, and Ravens test to give a more appropriate cases-to-items ratio (155:24) (Costello and Osborne, 2005).

Model fit. The fit of CFA and SEM models was assessed with several fit indices using conventional thresholds (Browne and Cudeck, 1992; Hu and Bentler, 1999; Albright and Park, 2009). Because the Chi-square fit index is overly sensitive for large degrees of freedom (Wheaton et al., 1977), the ratio of *χ*^2^/*df* is accepted as fit evidence if it has a statistically non-significant *p* > 0.05. The comparative fit index (CFI) indicates acceptable fit if it is above 0.90 and good fit above 0.95. We also included the more stable gamma hat index, interpreted in the same way as the CFI (Fan and Sivo, 2007). Both the root mean square error of approximation (RMSEA) and standardized root mean residual (SRMR) indicate acceptable fit if they are below 0.08 and good fit when below 0.05 (Browne and Cudeck, 1992; Hu and Bentler, 1999; Albright and Park, 2009).

## Results

### Measurement models

A four-factor structure with 20 items of the Swe-EAI showed acceptable fit to the data (*χ*^2^_(164)_ = 262.30, *χ*^2^/*df* = 1.60, *p* = 0.21; CFI = 0.88; gamma hat index = 0.93, RMSEA = 0.070, 90% CI [0.056, 0.083]; SRMR = 0.08). The relatively low CFI value probably arises from model complexity (Fan and Sivo, 2007) and can be discounted in light of the more robust values for the more stable indices (i.e., gamma hat, SRMR). This administration of the Swe-EAI had comparable results to those seen in previous studies (Inzunza, 2015b; Inzunza et al., 2019).

Table 1 below shows descriptive scale statistics, as well as three estimates of reliability for the two empathy subconstructs Affective Response (AR) and Perspective-Taking (PT) showing similar information concerning scale characteristics.

**Table 1:** Scale descriptive information.

Scale	Reliability indices			Descriptive		
	Alpha	Omega	Raykov rho	*M*	SD	SEM
AR	0.70	0.71	0.67	26.13	3.74	2.05
PT	0.65	0.66	0.63	27.66	3.07	1.81
IMT Memory	0.68	0.60	0.66	2.10	2.26	1.28
IMT Intention	0.57	0.53	0.55	3.99	2.47	1.62
Eyes	0.52	0.51	0.49	27.49	3.52	2.44
Ravens odd	0.77	0.74	0.75	10.23	3.40	1.63
Ravens even	0.78	0.79	0.67	8.19	3.45	1.62

The Swe-IMT worked as intended in its shorter version. The mean number of errors was almost twice as high in the items measuring intentions compared to items measuring memory, suggesting that the ToM component is a more demanding skill (Table 1). The correlation between the number of errors was similar to previous studies (Spearman’s *ρ* = 0.47, *p* < 0.01; Pearson *r* = 0.61, *p* < 0.01). The scale estimates of reliability were lower than normally expected, but high scale reliabilities may reflect item homogeneity (Cattell and Tsujioka, 1964), which is not the case for the IMT Intention and Memory scales that are more heterogeneous in content. These values are deemed sufficient for research purposes.

The Eyes test mean (Table 1) is within the mean ranges reported previously. The conventional estimates of scale reliability were low, but this is expected with binary-scored items. Nevertheless, the SEm was relatively small, indicating that score differences of ≥7 are statistically significant at a 95% confidence level.

The mean score for the odd Ravens items was slightly higher (*d* = 0.60, 90% CI = 0.27–0.92) than the mean score of the even items (Table 1). However, the estimates of reliability were robust, producing quite small SEm relative to means.

### Scale inter-relationships

The factor inter-correlations for participants with complete data showed that few of the linear relations had statistical significance (Table 2). Of interest here is the weak positive relationship of the two EAI and two IMT scales to the Eyes test. In contrast, only the AR and IMT Intention scales had a similar strength of relationship with the Ravens scale. The within-construct correlations for the Swe-EAI were moderate and strong for the Swe-IMT scales.

**Table 2:** Statistically significant Pearson inter-correlations between the four instruments.

Construct and Scale	Swe-EAI		Swe-IMT			
	I	II	III	IV	V	VI
*Swe-EAI*						
I. Affective response (AR)						
II. Perspective-taking (PT)	0.34^**^					
*Swe-IMT*						
III. Intention	*ns*	*ns*				
IV. Memory	*ns*	0.18^*^	0.61^***^			
V. Eyes	0.18^*^	0.22^**^	0.16^*^	0.23^**^		
VI. Ravens	0.18^*^	*ns*	0.24^**^	*ns*	*ns*	

ns = not significant. ^*^p < 0.05, ^**^p < 0.01, and ^***^p < .001.

**Figure 1:**
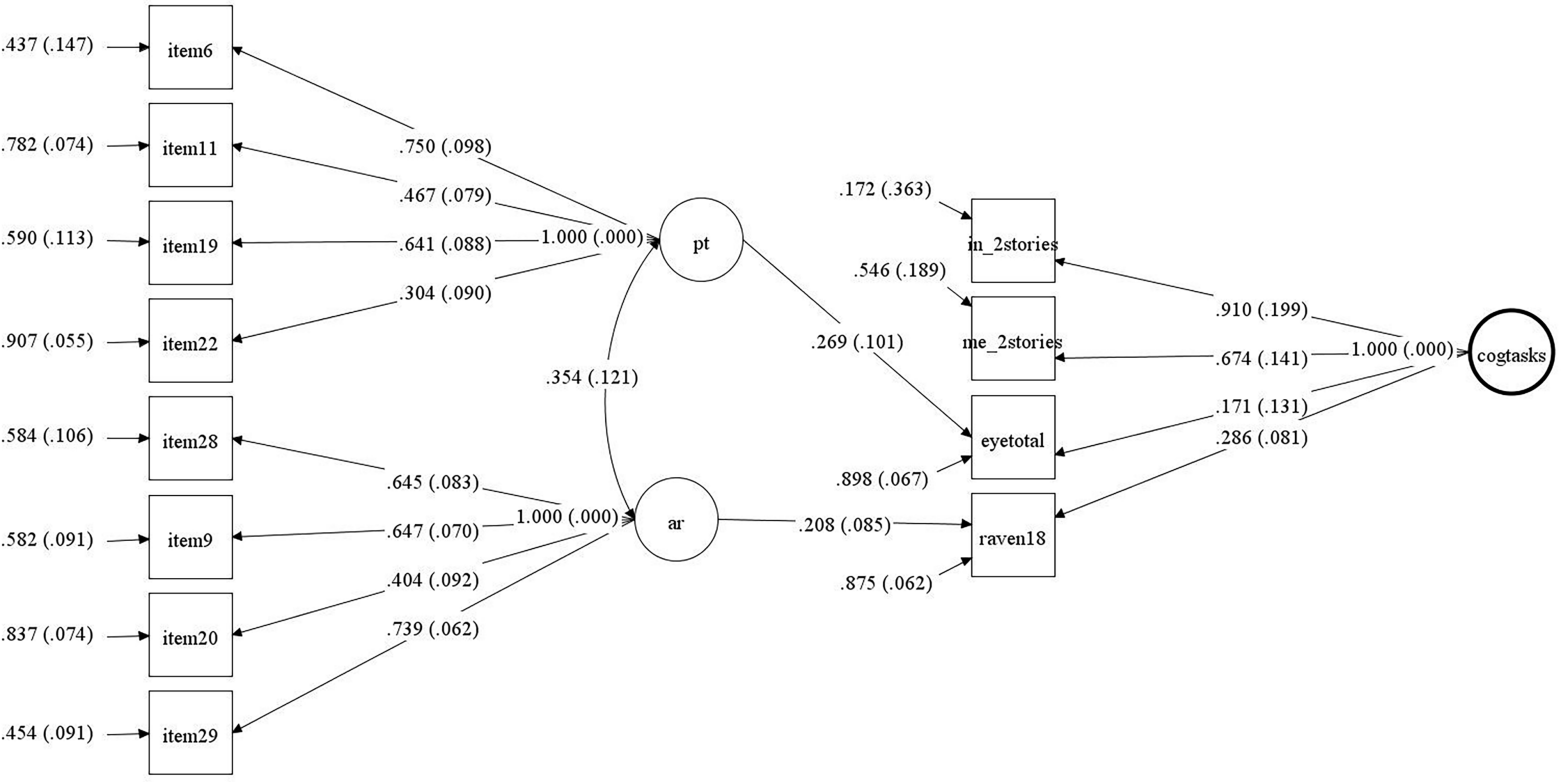
Latent variable model representing tested relations between self-reported an task measures. pt. = perspective-taking; ar = affective response; cogtasks = cognitive tasks; eyetotal = Eyes test; Raven 18 = Ravens fluid test; in_2stories = IMT intentions; and me_2stories = IMT memory.

The fully correlated model, with six items each for PT and AR, had poor fit to the data (*χ*^2^_(93)_ = 178.34, *χ*^2^/*df* = 1.97, *p* < 0.01; CFI = 0.79; gamma hat = 0.93., RMSEA = 0.081, 90% CI [0.063, 0.098]; SRMR = 0.079). Trimming two items each from PT and AR for weak loadings generated a better fitting model (*χ*^2^_(43)_ = 62.34, *χ*^2^/*df* = 1.82, *p* < 0.05; CFI = 0.933; gamma hat = 0.97, RMSEA = 0.058, 90% CI [0.024, 0.085]; SRMR = 0.055). Instead of treating each scale as an independent construct, the three measures reflecting processes within the cognitive dimension (i.e., Ravens, IMT Intention, and IMT Memory) were treated as indicators of a latent trait for cognitive processes measured as tasks. Given that the Eyes test is meant to reflect and thus indirectly measure the mental state of an individual, it was also positioned as an indicator of overall cognitive processes measured with cognitive tasks. Rather than simply correlate the scales, both PT and AR were situated as predictors of the three cognitive processes and the Eyes test to test the hypotheses. That model had good fit (*χ*^2^_(51)_ = 78.35, *χ*^2^/*df* = 1.54, *p* = <0.05; CFI = 0.91; gamma hat = 0.97, RMSEA = 0.058, 90% CI [0.030, 0.083]; SRMR = 0.063.) and showed that PT had a modest standardized regression weight to the Eye test and AR had a weaker regression weight to Ravens (Figure 1).

## Discussion

The aim of the present study was to shed light on the relation between measures of empathy and higher-order constructs of cognitive ability. There are few studies within this area and even fewer dealing with the specific population investigated in this study, that is police students. Within the police profession, it is often emphasized that to function well in daily work routines it is important to be prepared for complex and emotionally challenging situations with citizens in different contexts (Romosiou et al., 2019). Some examples of such situations include de-escalating conflict situations (Abanonu, 2018), interacting with victims of crime at crime scenes (Maddox et al., 2011), interrogating individuals with hidden agendas (Strömwall and Willén, 2011), or conducting investigative interviews of victims (Jakobsen, 2021). When facing these complex contact situations, there are several processes at work, both cognitive and emotional, and it is valuable to understand better how they are related. This knowledge is certainly necessary when seeking to implement different programs for improving police performance, or when allocating police officers for different missions.

In our study, we found that the psychometric properties from each instrument were similar to the ones seen in previous research. The multi-dimensional self-reported instrument of empathy Swe-EAI could differentiate between the two areas of cognitive and affective dimensions (Inzunza, 2015b; Inzunza et al., 2019). All task instruments developed to be used as proxies for the different abilities of interest also worked in a similar way as in previous studies (see Bors and Stokes, 1998; Kinderman et al., 1998; Baron-Cohen et al., 2001; Söderstrand and Almkvist, 2012; Inzunza et al., 2019).

After conducting several analyses, the findings that emerge from our data indicate that the affective dimension (AR) and the cognitive dimension (PT) do predict performance in different tasks (Figure 1). The preset hypotheses were not fully supported, they were supported to some level. Regarding the relation between subconstructs of empathy and cognitive ability, there is a significant prediction from the affective dimension, affective response (AR), to the shortened version of the Raven fluid test, which is a proxy for fluid intelligence ability: this is in line with some of the associations found in previous studies (e.g., Guo et al., 2019). The second hypothesis, if there is a positive relation between the subconstructs of empathy and tasks, the results show that perspective-taking ability is associated with how well a test-taker will perform in the Eyes test, which is a proxy for the ability to understand the mental state of others.

Another finding of interest is the non-significant relation between fluid intelligence and the Eyes test, which concerns the ability of a respondent to correctly identify the emotional states of others (Baron-Cohen et al., 2001; Baker et al., 2014). There are multiple reasons for there not being a relation since fluid intelligence may be something distinct from where an individual shows interest in another individual in the sense of what they may be experiencing. With the chosen sample in this study, we found a relation between self-reported perspective-taking and the Eyes test but no relation between the Eyes test and the shortened Ravens test measuring fluid intelligence. This finding is in line with the theories suggesting that ToM is associated with cognitive empathy (Singer, 2006). Further, the standardized loading from perspective-taking was considerably greater than from the latent cognitive task ability, suggesting that the Eyes test reflected more of the ability to empathically relate to how another person feels rather than a strictly intellectual ability. This gives some support to the notion that such relation is probably not direct, or is so weak that it is not beyond mere chance. Although the memory part of the IMT had a weak correlation with perspective-taking (Table 1), the more robust latent variable model (Figure 1) showed that after controlling for measurement error this association was not greater than chance. The memory part of the IMT showed no correlation to the shortened Ravens test, which was an unexpected result considering that previous studies have shown a relationship between working memory and episodic memory and fluid intelligence (Fukuda et al., 2010; Brewer and Unsworth, 2012). The memory part from the IMT was developed to control for memory not being an issue when measuring the intention part of the test, and it may not be a developed memory test *per se*.

In practice, the findings indicate that it is important to consider the interest of future applicants to professions dealing with people since performing well in tasks requiring general cognitive ability is different from performing well in tasks requiring the understanding of the other. Programs focusing at developing performance requiring these abilities (see Darwinkel et al., 2013) may require a different pedagogical approach before moving into the actual task training. The findings are also transferable and valuable to closely related areas such as emotional intelligence where instruments have been developed to be used in leadership training within police organizations following the principle of policing by consent such as in the United Kingdom (McDowall et al., 2019).

### Limitations

Some of the limitations of this study stem from the small sample size of participants. It may be difficult to predict performance on different tasks. It would be valuable to conduct a similar study with a larger sample, including professionals from within the police organization, rather than just police students. Such a study might reveal different relations once police students gain experience in the field. More importantly, the current results have to be seen as exploratory and require replication to validate the observed relations. While the project team will be able to map cognitive and affective empathy to emotion recognition, intention detection, and working memory in future studies, obtaining information with fluid intelligence measures in similar contexts is more challenging. It may be possible to test these findings with a sample from the general population of university students but that is currently out of scope for the project. Furthermore, such a study would not provide robust information about the population of concern, that is the police.

Generalizability to other police educational organizations is limited by the fact of having collected only Swedish data; hence, replication studies in different international contexts are warranted. This study was conducted among police students, but findings of the structure of the EAI have been used in an evaluation with in-service police officers (Hansson et al., 2021). The current measures treat each of these interactive aspects of empathy and cognition separately. Perhaps, a dynamic integrated assessment, along the lines of the objective structured clinical examinations used in medical education (Sloan et al., 1995), would improve our understanding of how these constructs inter-relate.

### Conclusion

Our conclusions are that abilities beyond fluid intelligence are needed to understand emotional states and the intentions of others. Policing involves navigating complex and highly charged situations, where culturally specific knowledge of how individuals communicate is also required (Günthner, 2007)—this is a matter of increasing importance in the rapidly changing European demographic context (Ilie, 2019). In policing situations, officers need to remember relevant event information, express empathy, and identify possible intentions from fleeting facial expressions; clearly, this is a complex and dynamic process, and police training needs to prepare recruits for it as fully as possible, including in terms of empathy. Our findings which also are in line with previous studies are inherently valuable since there is higher expectancy from society that the police will act empathically and treat citizens professionally: while good performance in contact situations can have a weak positive effect on police trustworthiness, poor performance has a strong negative effect, essentially meaning that trustworthiness is hard to achieve but easy to lose (Oliveira et al., 2020). Thus, this study adds to our understanding of how we might improve the empathy of police students as they prepare to enter a very demanding profession.

## Data availability statement

The raw data supporting the conclusions of this article will be made available by the authors, on reasonable requests.

## Ethics statement

Ethical review and approval was not required for the study on human participants in accordance with the local legislation and institutional requirements. The patients/participants provided their written informed consent to participate in this study.

## Author contributions

MI, TS, and CW contributed to conception and design of the study. MI organized the database and wrote the first draft of the manuscript. MI and GB performed statistical analysis. GB, TS, and CW wrote sentences of the manuscript. All authors contributed to the article and approved the submitted version.

## Funding

This work is part of a series of studies investigating constructs of relevance in police training and education. It was funded by the Swedish Research Council via Grant [2016-04842].

## Conflict of interest

The authors declare that the research was conducted in the absence of any commercial or financial relationships that could be construed as a potential conflict of interest.

## Publisher’s note

All claims expressed in this article are solely those of the authors and do not necessarily represent those of their affiliated organizations, or those of the publisher, the editors and the reviewers. Any product that may be evaluated in this article, or claim that may be made by its manufacturer, is not guaranteed or endorsed by the publisher.

## References

[ref1] Abanonu R. (2018). De-escalating police-citizen encounters. S. Calif. Rev. Law Soc. Justice 27, 239–269.

[ref2] Albright J. J. Park H. M. (2009). Confirmatory Factor Analysis Using Amos, LISREL, Mplus, and SAS/STAT CALIS. Indianapolis, IN: The Trustees of Indiana University, pp. 1–85.

[ref3] Anderson J. C. Gerbing D. W. (1988). Structural equation modeling in practice–a review and recommended 2-step approach. Psychol. Bull. 103, 411–423. doi: 10.1037/0033-2909.103.3.411

[ref4] Annell S. Sjöberg A. Sverke M. (2014). Use and interpretation of test scores from limited cognitive test batteries: how g + Gc can equal g. Scand. J. Psychol. 55, 399–408. doi: 10.1111/sjop.12140, PMID: 25040205

[ref5] Arthur W. Jr. Day D. V. (1994). Development of a short form for the raven advanced progressive matrices test. Educ. Psychol. Meas. 54, 394–403. doi: 10.1177/0013164494054002013

[ref6] Baker C. A. Peterson E. Pulos S. Kirkland R. A. (2014). Eyes and IQ: a meta-analysis of the relationship between intelligence and ‘Reading the mind in the eyes’. Intelligence 44, 78–92. doi: 10.1016/j.intell.2014.03.001

[ref7] Baron-Cohen S. Bowen D. C. Holt R. J. Allison C. Auyeung B. Lombardo M. V.. (2015). The ‘reading the mind in the eyes’ test: complete absence of typical sex difference in~ 400 men and women with autism. PLoS One 10:e0136521. doi: 10.1371/journal.pone.0136521, PMID: 26313946 PMC4552377

[ref8] Baron-Cohen S. Wheelwright S. Hill J. Raste Y. Plumb I. (2001). The “Reading the mind in the eyes” test revised version: a study with normal adults, and adults with Asperger syndrome or high-functioning autism. J. Child Psychol. Psychiatry 42, 241–251. doi: 10.1111/1469-7610.00715 11280420

[ref111] Beauregard E. Rossmo D. K. Proulx J. (2007). A descriptive model of the hunting process of serial sex offenders: a rational choice perspective. J. Fam. Violence. 22, 449–463.

[ref9] Bloksgaard L. Prieur A. (2021). Policing by social skills: the importance of empathy and appropriate emotional expressions in the recruitment, selection and education of Danish police officers. Polic. Soc. 31, 1232–1247. doi: 10.1080/10439463.2021.1881518

[ref10] Bors D. A. Stokes T. L. (1998). Raven's advanced progressive matrices: norms for first-year university students and the development of a short form. Educ. Psychol. Meas. 58, 382–398. doi: 10.1177/0013164498058003002

[ref11] Brewer G. A. Unsworth N. (2012). Individual differences in the effects of retrieval from long-term memory. J. Mem. Lang. 66, 407–415. doi: 10.1016/j.jml.2011.12.009

[ref12] Browne M. W. Cudeck R. (1992). Alternative ways of assessing model fit. Sociol. Methods Res. 21, 230–258. doi: 10.1177/0049124192021002005

[ref13] Carpenter P. A. Just M. A. Shell P. (1990). What one intelligence test measures: a theoretical account of the processing in the raven progressive matrices test. Psychol. Rev. 97, 404–431. doi: 10.1037/0033-295X.97.3.404, PMID: 2381998

[ref14] Cattell R. B. Tsujioka B. (1964). The importance of factor-trueness and validity, versus homogeneity and orthogonality, in test scales. Educ. Psychol. Meas. 24, 3–30. doi: 10.1177/001316446402400101

[ref15] Charman S. (2019). Making sense of policing identities: the ‘deserving’and the ‘undeserving’in policing accounts of victimisation. Polic. Soc. 30, 81–97. doi: 10.1080/10439463.2019.1601721

[ref16] Costello A. B. Osborne J. W. (2005). Best practices in exploratory factor analysis: four recommendations for getting the most from your analysis. Pract. Assess. Res. Eval. 10, 1–9. doi: 10.7275/jyj1-4868

[ref17] Darwinkel E. Powell M. Tidmarsh P. (2013). Improving police officers’ perceptions of sexual offending through intensive training. Crim. Justice Behav. 40, 895–908. doi: 10.1177/0093854813475348

[ref18] Davis M. H. (1983). Measuring individual differences in empathy: evidence for a multidimensional approach. J. Pers. Soc. Psychol. 44, 113–126. doi: 10.1037/0022-3514.44.1.113

[ref112] Decety J. Jackson P. L. (2006). A social-neuroscience perspective on empathy. Curr. Dir. Psychol. Sci. 15, 54–58.

[ref19] Fan X. Sivo S. A. (2007). Sensitivity of fit indices to model misspecification and model types. Multivar. Behav. Res. 42, 509–529. doi: 10.1080/00273170701382864

[ref20] Fernández-Abascal E. G. Cabello R. Fernández-Berrocal P. Baron-Cohen S. (2013). Test-retest reliability of the ‘Reading the mind in the eyes’ test: a one-year follow-up study. Mol. Autism. 4, 1–6. doi: 10.1186/2040-2392-4-33 24020728 PMC3848772

[ref21] Fukuda K. Vogel E. Mayr U. Awh E. (2010). Quantity, not quality: the relationship between fluid intelligence and working memory capacity. Psychon. Bull. Rev. 17, 673–679. doi: 10.3758/17.5.673, PMID: 21037165 PMC3050565

[ref22] Gore N. G. Barnes-Holmes Y. Murphy G. (2010). The relationship between intellectual functioning and relational perspective-taking. Int. J. Psychol. Psychol. Ther. 10, 1–17.

[ref23] Günthner S. (2007). “Intercultural communication and the relevance of cultural specific repertoires of communicative genres” in Handbook of Intercultural Communication. eds. Kotthoff H. Spencer-Oatey H. (Berlin, New York, NY: Mouton de Gruyter), 127–152.

[ref24] Guo Q. Sun P. Cai M. Zhang X. Song K. (2019). Why are smarter individuals more prosocial? A study on the mediating roles of empathy and moral identity. Intelligence 75, 1–8. doi: 10.1016/j.intell.2019.02.006

[ref25] Hansson J. Inzunza M. Stjerna Doohan I. (2021). The Norwegian Police’s Use of Conducted Energy Weapons: A Scientific Evaluation of the CEW Trial 2019–2020. Umeå: Umeå universitet.

[ref26] Hu L.-T. Bentler P. M. (1999). Cutoff criteria for fit indexes in covariance structure analysis: conventional criteria versus new alternatives. Struct. Equ. Model. 6, 1–55. doi: 10.1080/10705519909540118

[ref29] Ilie O.-A. (2019). The intercultural competence. Developing effective intercultural communication skills. Int. Conf. Knowledge Organ. 25, 264–268. doi: 10.2478/kbo-2019-0092

[ref30] Inzunza M. (2015a). Empathy from a police work perspective. J. Scand. Stud. Criminol. Crime Prev. 16, 60–75. doi: 10.1080/14043858.2014.987518

[ref31] Inzunza M. (2015b). Adaptation and development of the empathy assessment index (EAI). Int. J. Comp. Appl. Crim. Just. 39, 239–255. doi: 10.1080/01924036.2014.989245

[ref32] Inzunza M. (2022). The significance of victim ideality in interactions between crime victims and police officers. Int. J. Law Crime Justice 68:100522. doi: 10.1016/j.ijlcj.2021.100522

[ref33] Inzunza M. Stenlund T. Wikström C. (2019). Measuring perspective taking among police recruits. Policing: an. Int. J. 42, 725–738. doi: 10.1108/PIJPSM-09-2018-0129

[ref34] Jakobsen K. K. (2021). Empathy in investigative interviews of victims: how to understand it, how to measure it, and how to do it? Police Pract. Res. 22, 1155–1170. doi: 10.1080/15614263.2019.1668789

[ref35] Jolliffe D. Farrington D. P. (2006). Development and validation of the Basic Empathy Scale. J. Adolesc. 29, 589–611. 16198409 10.1016/j.adolescence.2005.08.010

[ref36] Kane M. (2010). Validity and fairness. Lang. Test. 27, 177–182. doi: 10.1177/0265532209349467

[ref37] Kinderman P. Dunbar R. Bentall R. P. (1998). Theory-of-mind deficits and causal attributions. Br. J. Psychol. 89, 191–204. doi: 10.1111/j.2044-8295.1998.tb02680.x

[ref38] Launay J. Pearce E. Wlodarski R. van Duijn M. Carney J. Dunbar R. I. (2015). Higher-order mentalising and executive functioning. Personal. Individ. Differ. 86, 6–14. doi: 10.1016/j.paid.2015.05.021, PMID: 26543298 PMC4630865

[ref39] Leclerc B. (2017). “Crime scripts” in Environmental Criminology and Crime Analysis. eds. Wortley R. Townsley M.. 2nd ed (Oxon: Routledge), 119–141.

[ref40] Maddox L. Lee D. Barker C. (2011). Police empathy and victim PTSD as potential factors in rape case attrition. J. Police Crim. Psychol. 26, 112–117. doi: 10.1007/s11896-010-9075-6

[ref41] Martingano A. J. Konrath S. (2022). How cognitive and emotional empathy relate to rational thinking: empirical evidence and meta-analysis. J. Soc. Psychol. 162, 143–160. doi: 10.1080/00224545.2021.1985415, PMID: 35083952

[ref42] McDowall A. Brown J. Gamblin D. (2019). Assessing emotional intelligence of graduate probationer police officers: a UK pilot study. Policing J. Policy Pract. 14, 104–118. doi: 10.1093/police/paz039

[ref43] Murphy B. A. Lilienfeld S. O. (2019). Are self-report cognitive empathy ratings valid proxies for cognitive empathy ability? Negligible meta-analytic relations with behavioral task performance. Psychol. Assess. 31, 1062–1072. doi: 10.1037/pas0000732, PMID: 31120296

[ref44] Muthén L. K. Muthén B. O. (1998-2017). Mplus User’s Guide. 8th *Edn.* Los Angeles, CA: Muthén & Muthén.

[ref45] Oliveira T. R. Jackson J. Murphy K. Bradford B. (2020). Are trustworthiness and legitimacy ‘hard to win, easy to lose’? A longitudinal test of the asymmetry thesis of police-citizen contact. J. Quant. Criminol. 37, 1003–1045. doi: 10.1007/s10940-020-09478-2

[ref113] Perez D. W. (2010). Paradoxes of Police Work. New York, NY: Cengage Learning.

[ref46] Rahr S. Rice S. K. (2015). “From warriors to guardians: recommitting American police culture to democratic ideals,” in New Perspectives in Policing (Laurel, MD: National Institute of Justice, U.S Department of Justice).

[ref47] Rakoczy H. Harder-Kasten A. Sturm L. (2012). The decline of theory of mind in old age is (partly) mediated by developmental changes in domain-general abilities. Br. J. Psychol. 103, 58–72. doi: 10.1111/j.2044-8295.2011.02040.x, PMID: 22229774

[ref48] Richell R. A. Mitchell D. G. Newman C. Leonard A. Baron-Cohen S. Blair R. J. R. (2003). Theory of mind and psychopathy: can psychopathic individuals read the ‘language of the eyes’? Neuropsychologia 41, 523–526. doi: 10.1016/s0028-3932(02)00175-6, PMID: 12559146

[ref114] Romosiou V. Brouzos A. Vassilopoulos S. P. (2019). An integrative group intervention for the enhancement of emotional intelligence, empathy, resilience and stress management among police officers. Police Pract. Res. 20, 460–478.

[ref115] Singer T. (2006). The neuronal basis and ontogeny of empathy and mind reading: review of literature and implications for future research. Neurosci. Biobehav. Rev. 30, 855–863. 16904182 10.1016/j.neubiorev.2006.06.011

[ref49] Skogan W. G. (2006). Asymmetry in the impact of encounters with police. Polic. Soc. 16, 99–126. doi: 10.1080/10439460600662098

[ref50] Sloan D. A. Donnelly M. B. Schwartz R. W. Strodel W. E. (1995). The objective structured clinical examination. The new gold standard for evaluating postgraduate clinical performance. Ann. Surg. 222, 735–742. doi: 10.1097/00000658-199512000-00007, PMID: 8526580 PMC1235022

[ref51] Söderstrand P. Almkvist O. (2012). Psychometric data on the eyes test, the faux pas test, and the Dewey social stories test in a population-based Swedish adult sample. Nordic Psychol. 64, 30–43. doi: 10.1080/19012276.2012.693729

[ref52] STATA (2020). Stata Statistical Software: Release 16.1. College Station, TX: STATA Corp..

[ref116] Strömwall L. A. Willén R. M. (2011). Inside criminal minds: Offenders’ strategies when lying. J. Investig. Psychol. Offender Profiling 8, 271–281.

[ref53] Van Prooijen J.-W. van Dijk E. (2014). When consequence size predicts belief in conspiracy theories: the moderating role of perspective taking. J. Exp. Soc. Psychol. 55, 63–73. doi: 10.1016/j.jesp.2014.06.006

[ref54] Vellante M. Baron-Cohen S. Melis M. Marrone M. Petretto D. R. Masala C.. (2013). The ‘Reading the mind in the eyes’ test: systematic review of psychometric properties and a validation study in Italy. Cogn. Neuropsychiatry 18, 326–354. doi: 10.1080/13546805.2012.721728, PMID: 23106125 PMC6345369

[ref55] Wheaton B. Muthén B. Alwin D. F. Summers G. F. (1977). Assessing reliability and stability in panel models. Sociol. Methodol. 8, 84–136. doi: 10.2307/270754

[ref56] Yan Z. Hong S. Liu F. Su Y. (2020). A meta-analysis of the relationship between empathy and executive function. Psychol. J. 9, 34–43. doi: 10.1002/pchj.311, PMID: 31394592

